# Influence of different adhesion strategies on glass fiber post retention

**DOI:** 10.4317/jced.60452

**Published:** 2023-08-01

**Authors:** Ana-Michelle-Oliveira Nadler, Evair-Josino da Silva, Paulo-Cardoso Lins-Filho, Marlon-Ferreira Dias, Renata-Pedrosa Guimarães, Claudio-Heliomar-Vicente da Silva, Sérgio-dos Santos Silva, Anderson-Stevens-Leonidas Gomes

**Affiliations:** 1 PhD Student of the Dentistry Post-Graduate Program, Universidade Federal de Pernambuco, Recife, Brazil; 2 PhD Student of the Post-Graduate Program in Oral Rehabilitation, Universidade Estadual de São Paulo – UNESP, Araraquara, Brazil; 3 Professor of the Dentristry Department, Universidade Federal de Pernambuco, Recife, Brazil; 4 Head of the Laboratory technicians of the Department of Physics, Universidade Federal de Pernambuco, Recife, Brazil; 5 Professor of the Dentistry Post-Graduate Program, Universidade Federal de Pernambuco, Recife, Brazil; 6 Professor of the Department of Physics, Universidade Federal de Pernambuco, Recife, Pernambuco, Brazil

## Abstract

**Background:**

Failures in glass fiber post (GFP) retention may be associated with low adhesion achieved in root dentin.

**Material and Methods:**

55 single-rooted premolars were endodontically treated and distributed according to different adhesion strategies (n=11): G1: RelyX ARC (3M ESPE; etch-rinse strategy); G2: Relyx Ultimate (3M ESPE; etch-rinse strategy); G3: AllCem (FGM; etch-rinse strategy); G4: Relyx Ultimate (3M ESPE; self-etching strategy); G5: RelyX U200 (3M ESPE; self-adhesive strategy). For Bonding Strength (BS) analysis, the roots were sectioned in slices (1.0mm thickness) corresponding to each root third and submitted to push-out test. The type of failure was assessed by scanning electron microscopy (SEM).

**Results:**

The highest BS averages were found in G2 and G3. However, in the middle and apical root thirds, G3 showed statistically similar results to G4 and G5. In the cervical and middle third, G1 was statistically similar to G4 and G5. The mixed type of failure was the most common in all groups.

**Conclusions:**

Self-etching (G4) and self-adhesive resin (G5) cements, showed similar BS results of immediate bonding in the cementation of GFP compared to conventional resin cements (G1, G2, G3).

** Key words:**Dental Cements, Dentin-Bonding Agents, Post and Core Technique, Dental Bonding.

## Introduction

The increased demand for aesthetic adhesive rehabilitation treatments has driven the development of materials that meet aesthetic and functional requirements to ensure the longevity of direct and indirect restorations (1). The rehabilitation of endodontically treated teeth follows this trend. Intraradicular retainers that present mechanical characteristics similar to the dental structure have the ability to reduce stresses transmitted to the dental remnant, minimising the risk of fractures and reducing treatment costs compared to molten metal cores (2).

Intraradicular retainer’s failures may be associated with low adhesion achieved in root dentin, this may occur due to inadequate biomechanical preparation, permanence of filling material in the root canal, type of cement used, incorrect cement handling, peculiar morphological characteristics of the dentinal tissue, or the complexity and sensitivity of the adhesive and cementation technique (3,4). To avoid failure in rehabilitation treatments, the choice of the ideal luting material by the professional must be based on three fundamental aspects: simplicity of the technique, cost-benefit ratio and available scientific evidence (5). However, this decision is impaired by the great variability of protocols and materials available on market, combined with the scarcity of studies that include, in equal numbers, the vast majority of these.

The most suitable materials for luting glass fiber posts (GFP) into the root canal are resin cements, which can be classified into three types according to their polymerization method: photoactivated, chemically activated, and dual (6). In addition, cements can adhere and interact with the radicular dentin surface in different ways. According to the bonding mechanism, they can be classified as conventional, self-etching, and self-adhesive (7).

Self-adhesive cements were designed with the purpose of overcoming some of the limitations of both conventional and self-etch resin cements (8). Self-adhesive cements do not require any pretreatment of the tooth substrate: Once the cement is mixed, the application is performed in a single clinical step (9,10). These cements have an adhesion mechanism through micromechanical retention and chemical bonding to the dental structure (7). A systematic review of *in vitro* studies has shown that self-adhesive resin cements are effective in retaining GFP into root canals (1).

To study the influence of different adhesion strategies on GFP retention, it is required a thorough analysis due to the complexity of the root dentin and post interface, since it involves not only the interaction between cement and dentin but also between cement and the post itself (8). This way, in addition to evaluate the bond strength (BS) values trough mechanical tests, it is necessary to observe which type of failure may occurred on the interface, those failures may be adhesive between dentin and cement or between cement and post, or cohesive, represented by fractures in dentin, cement and post. Under masticatory forces, it is also common for more than one type of failure to occur simultaneously, and these are called mixed failures (12). Both analyses, between the type of failure and mechanical assay, will produce results in an experimental study with more clinical significance.

This study aimed to evaluate the performance of different resin cements, including conventional, self-etching, and self-adhesive, in the bond strength of GFP along the root dentin, through the mechanical push-out bond strength test. In addition, the integrity of the hybrid layer and the types of failures that occurred at the adhesive interface after the mechanical test were evaluated through scanning electron microscopy (SEM). The null hypothesis admits that the type of cement and depth of the roots influence the bond strength of GFP to dentin.

## Material and Methods

This study was submitted and approved by the local Research Ethics Committee (protocol n. 740.915) and is in accordance with the Declaration of Helsinki.

Fifty-five single-rooted extracted human premolars with a length of >15mm were selected. The eligible tooth had to have a single root canal, no apical curvature, no caries lesion or root crack, and no previous endodontic treatment. The teeth were cleaned and stored for seven days in 0.5% chloramine T solution under refrigeration for disinfection. The samples were then kept in sterile saline at 37°C, replaced every seven days.

-Root Canal Preparation

The crowns were then sectioned with a double-sided diamond disc and discarded to standardize the root length by 15 mm. All root canals were instrumented by the same operator. Canal patency was established with a 35-K size file (Dentsply Maillefer). Endodontic instrumentation was performed with a Gates Glidden drill no. 1 to 4 (Dentsply-Maillefer, Tulsa, USA) and irrigation with 2.5% sodium hypochlorite. Roots were dried with paper points (Dentsply Maillefer) and then filled with gutta percha #35 and FF points (Dentsply-Maillefer, Tulsa, USA), cemented with eugenol-free cement (Sealer 26 / Dentsply Caulk, Millford, USA). The apex and coronary portion were protected from direct contact with moisture with wax and the samples were stored in sterile saline at 37°C for one week.

-Post Space Preparation and Experimental Groups

The root canals were prepared with low-speed Largo drill n° 2 to 4 (Dentsply Maillefer, Tulsa, USA), with a cursor positioned at 10.0 mm and finished with the drill corresponding to the White post DC pin n°0.5 (FGM, Joinville, Santa Catarina, Brazil). Then the root canals were irrigated with saline. The drills were replaced every 10 preparations. After removing the filling material, it was obtained a post space 10.0 mm long and 5.0 mm short of the root apex.

After post space preparation, roots were randomly assigned to one of the five groups (n=11) according to the resin cement used (Table 1), the GFP (White posts DC no. 0.5 - FGM, Joinville, Santa Catarina, Brazil) were placed in the root canals according to manufacturer’s recommendations (Table 2).

**Table 1:**
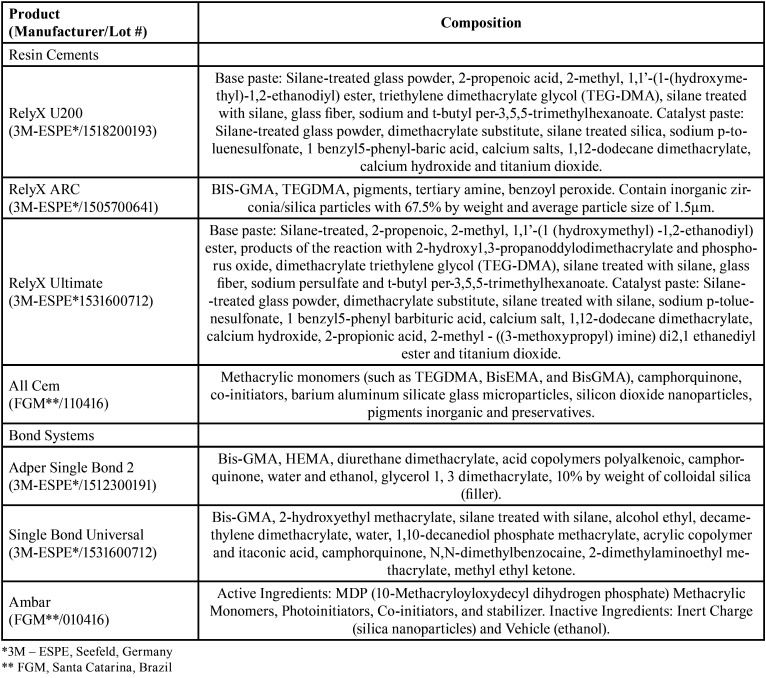
Composition of the used materials according to manufacturers.

**Table 2:**
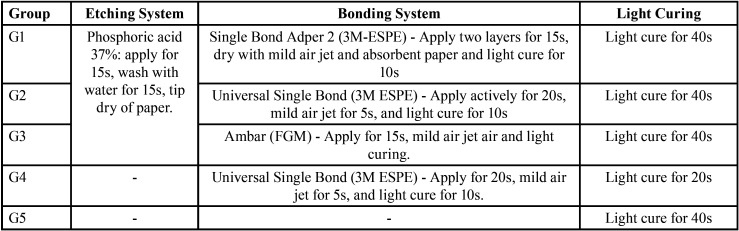
Applying procedures for the evaluated materials.

For all roots a Centrix syringe (needle-like tip) was used to insert the cement into the root canal following the manufacturers’ recommendations. Before cementation, each GFP was cleaned with 70% alcohol, dried, and a silane agent (Angelus, Paraná, Brazil) was applied according to the manufacturer’s instructions. In all groups, except for G5, the adhesives were applied with an applicator brush (Microaplicador KG Brush - KG SORENSEN, Brazil), to ensure that the bonding agent penetrated the entire root canal.

The photoactivation procedures were performed with a LED (Light Emitting Diode) device (Radii Cal - SDI, Victoria, Australia) with a light intensity of 1200mW/cm2, measured by a radiometer device (Hilux Ledmax, serial M4063022 - Benlioglu Dental Inc., Ankara, Turkey), and standardized at all stages. During photopolymerization, the cervical portion of the roots received a layer of aluminum foil, leaving exposed only the diameter of the tooth root to simulate photoactivation as it occurs in the oral cavity. The roots were then stored in sterile saline at 37°C for one week.

Roots were sectioned perpendicularly to their long axis in slices of 1.0 mm (± 0.2 mm) with a precision cutter (Elsaw - São Carlos, So Paulo, Brazil) in the cervical (C), middle (M) and apical thirds (A), to acquire samples for the BS test push-out test and SEM analysis. The sample calculation, estimated at 32 specimens for each resin cement, was carried out with the G*Power program version 3.1.9.2 considering an interval of confidence of 95% and 5% error.

-Pre-test Structural Analysis

Two specimens (cervical and apical) of one tooth from each group were used for analysis in the high resolution Tecscan Mira 3 SEM (TECSCAN, Pennsylvania, USA, nominal resolution 50nm). To remove the dentinal sludge generated by friction of the diamond discs in the roots section procedure, the fragment destined for the evaluation of the hybrid layer was subjected to etching with 37% phosphoric acid for 30 seconds, washing, drying, and deproteinization of the dentin with 2.5% sodium hypochlorite for 2 minutes. This procedure was followed by ultrasonic cleaning for 10 minutes to remove possible debris from the surface of the samples.

The samples were dehydrated by immersion in ascending degrees of acetone: 30% (10 min.); 50% (10 min.); 70% (20 min.); 90% (20 min.) and 100% (30 min.). After dehydration, the specimens were subjected to a drying process to evaporate all the solvent in a hothouse for 24 hours and kept in a silica gel desiccator with blue indicator.

After drying, the specimens were mounted on a metallic stub, a silver paint was applied to the side of the samples to improve electron beam conduction, and then the specimens were sputter coated with gold (Desk II - Denton Vacuum, Moorestown, Nova Jersey) with a current of 40 mA and a coverage time of 90 seconds for observation in the high-resolution SEM through the capture of secondary electrons.

For qualitative assessment of the adhesive interface and the hybrid layer through SEM, the following aspects were observed: a) uniformity of the hybrid layer along the entire length of the adhesive interface in the root thirds and absence of cracks and bubbles; b) presence of cracks in the interface: between the post and the cement; inside the cement layer; between the cement and the adhesive layer; inside the adhesive layer; and between the adhesive layer and dentin.

-Push-out Bonding Strength Test

**Figure 1:**
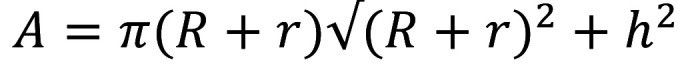
Formula.

The central slices of each third were considered for the test. Measurements, in millimetres, of the height (h) of the root canal of each segment, and measurements of the cervical diameter (R x 2) and the apical diameter (r x 2) were obtained with the aid of a digital caliper (LEE TOOLS, Ottawa, Canada). With these measurements, the bonding surface area (A) was calculated using the formula of a conical frustum, (Fig. 1): (12).

In the formula “π” it represents the constant 3.1416; ‘R’ is the largest radius in the cervical portion of the sample; ‘r’ the smallest radius of the root canal in the apical portion; ‘h’ is the thickness of the sample.

The specimens were submitted to the push-out extrusion compression test in a universal EMIC testing machine (São José dos Pinhais, Paraná, Brazil). A compressive load (2.000kg) was applied using a cylindrical diameter plunger (1 mm) at a constant speed of 0.5 mm/min in an apical coronal direction until the post was dislodged. The plunger was placed at the centre of each sample, directly in contact with the post fiber. Push-out bond strengths (MPa) were calculated for each specimen from the maximum force required to dislodge the post from the bonding surface area (A).

-Characterization of the type of failure through SEM

All samples were evaluated with a low resolution scanning electron microscope (TM 1000 HITACHI, São Paulo, Brazil). Representative areas were photographed at 80X and 120X magnification (nominal resolution 300nm). The images were evaluated by an experienced operator, which was blinded to the experimental groups. To evaluate the type of failure, the following classification was adopted: 1. Adhesive failure in the dentin-cement interface; 2. Adhesive failure in the cement-post interface; 3. Cohesive failure in cement; 4. Cohesive post failure; 5. Cohesive failure in dentin; 6. Mixed failure (when co-existing in the same specimen, adhesive and cohesive failure of any type).

-Statistical Analysis

Data were submitted to statistical analysis, all tests were applied considering an error of 5% and the confidence interval of 95%, and the analyzes were carried out using Statistical Package for the Social Sciences -SPSS / IBM software version 23.0 (SPSS Inc. Chicago, IL, USA). For the bond strength data, the means and standard deviations were calculated. The normality of the data was verified using the Shapiro-Wilk test. The groups were compared using the ANOVA test with Tamhane post hoc for comparison between cements and Bonferroni for comparison between root thirds. To determine the statistical significance of the types of adhesive failures, Fisher’s exact test was performed.

## Results

The megapascal averages (MPa) of the BS of each experimental group per third are shown in Table 3. In the comparison between the groups, the highest BS averages were found in G2 and G3. However, in the middle and apical thirds, G3 showed results statistically similar to G4 and G5. In the cervical and middle thirds, G1 was statistically similar to G4 and G5. In the comparison between the root thirds, higher values of adhesion were observed in the apical third for the groups G2 (13.79 MPa), G1 (13.49 MPa) and G3 (10.72 MPa). According to the Bonferroni multiple comparison test in the comparison between the thirds of the roots, within each cement group, significant differences were observed between the apical third and the other two thirds of the roots in the G1 and G4 groups, and between the thirds of the cervical and apical roots in G3.

**Table 3:**
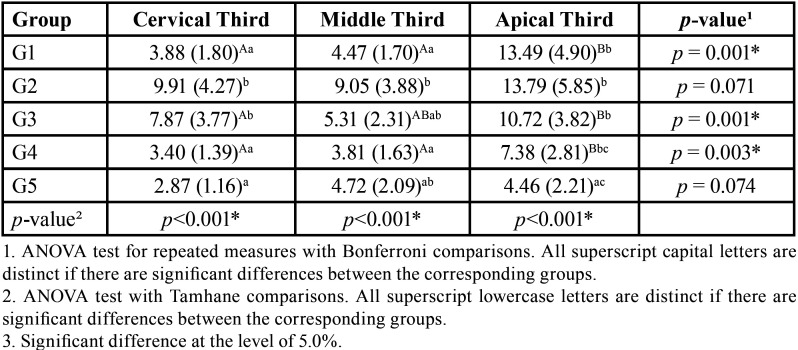
Mean (MPa) and standard deviation (SD) of the bond strength through push-out test according to the type of cement and root third.

**Figure 2:**
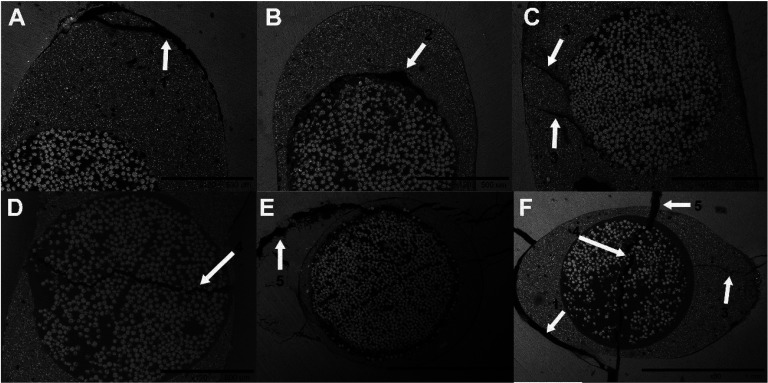
Photomicrographs obtained by low-resolution SEM technique, characterizing the types of failure: (A) Adhesive failure in the dentin-cement interface - arrow 1; (B) Adhesive failure in the cement-post interface – arrow 2; (C) Cohesive failure in resin cement – arrow 3; (D) Cohesive post failure – arrow 4; (E) Cohesive failure in dentin – arrow 5; (F) Mixed failure – combination in the same specimen, adhesive and cohesive failure of any type.

The SEM findings showed that three of the six types of failure adopted for this study were recorded: adhesive failure at the dentin-cement interface, cohesive failure in dentin and mixed failure (Fig. 2). However, due to the occurrence of only five cases of cohesive failure in dentin among the samples, it was excluded from the statistical analyses. Table 4 shows the frequencies and analysis of the types of failure by group for each root third. Mixed failure was more frequent than dentin-cement adhesive failure in all the root thirds, with percentages ranging from 66% (cervical) to 81.2% (apical), however, without significant differences (*p*> 0.05) between groups for each root third.

**Table 4:**
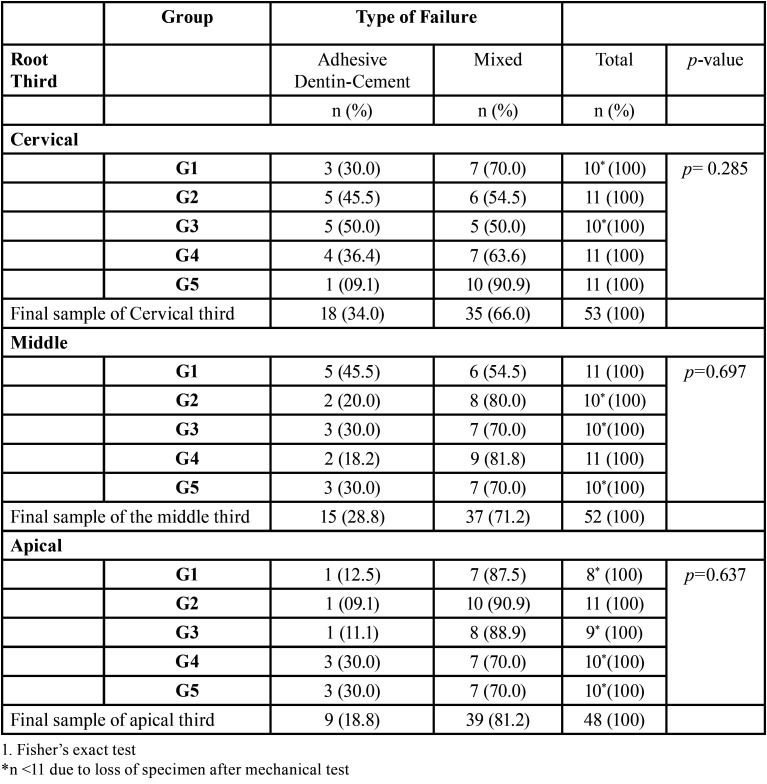
Frequencies of failure type according to root third and cement.

**Figure 3:**
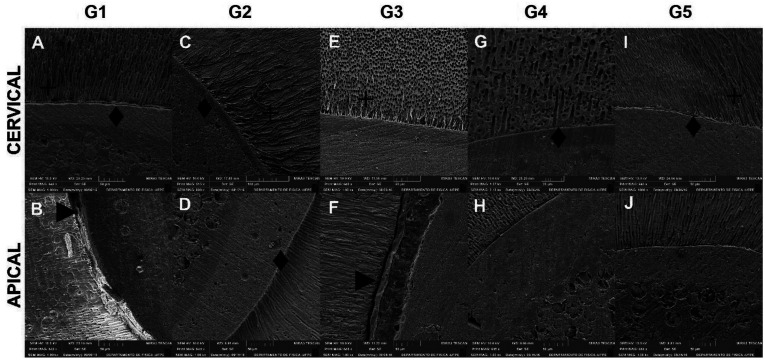
Characterization of the cement/dentin interface in SEM. The first line shows images of the tooth/cement interface at the cervical third of the root. The second line represents the image in the apical third. Each column represents the different types of cements tested. The symbols indicate: (♦) hybrid layer; (+) resin tags and (►) gaps, respectively.

The photomicrographs representative of the morphological patterns of the dentin-cement-post interface of the groups studied are shown in Figure 3. In images A and B, corresponding to G1, it is possible to observe the most regular cement-dentin interface in the cervical third. Images C and D (G2) showed cement-dentin interfaces with a clear hybrid layer, with the presence of resinous tags in the two thirds analysed. In the E (G3) image, there is also a clear hybrid layer with the formation of resinous extensions; however, in the apical third, there is a gap between adhesive and dentin (F). In the other groups, G4 and G5, although there is a good interaction between cement and dentin, there is an absence of resinous tags.

## Discussion

According to the results obtained, the null hypothesis was rejected since there was influence of the type of cement and the root depth on the bond strength of glass fiber post to dentin.

GFP technology extended the benefits of adhesive techniques to the rehabilitation of endodontically treated teeth, which favoured a significant reduction in root fractures, allowed better use of the dental remnant and restored tooth function with good aesthetic results (13). However, establishing an effective adhesion within the root canal is still a clinical challenge, since it is directly related to the quality of the interaction between the luting agent and dentin (14). Some factors such as dentin morphology, characteristics of the adhesive system, resin cement and its cure inside the root canal, can interfere in this interaction along the root canal walls, affecting post retention and causing its early displacement (14,15).

The highest values of BS were observed in the apical third of the root canals in accordance with a previous finding (7). These results may be associated with the lower degree of polymeric conversion or the slower polymerization of dual cements in the apical third, which can lead to less contraction stress and reduced effects of C factors (16). The type of post used is also an important factor, while once the intraradicular preparation is finished with a drill corresponding to the diameter of the post, the thickness of the cementation line decreases in the third apical, therefore the resistance to friction to post displacement increases (12). The role of the thickness of the cementation line on the BS of GFP is quite relevant and due to this fact, some studies suggested the use of anatomical posts or the association of multiple GFP to improve the BS in the cervical third where the cementation line tends to be thicker (17).

It was observed a good performance of conventional dual resin cements (G1, G2 and G3) that obtained high values of BS, which is probably related to the dentin demineralization pattern produced by phosphoric acid and, simultaneously, better bond infiltration on dentin (18). Photomicrographs obtained by low-resolution SEM (Fig. 2) reinforce this hypothesis, as, for these cements, especially the RelyX Ultimate Conventional, a more uniform hybrid layer and more regular dentin - cement - post interfaces were observed.

The RelyX Ultimate resin cement showed a different behaviour when used in different protocols. Although the manufacturer indicates its use under conventional or self-etching techniques, the result of the Single Bond Universal adhesive system (self-etch) when used without the use of phosphoric acid, in this work, revealed the lowest retention values. The SEM images revealed absence of resinous tags at the dentin-adhesive interface of this system, in accordance with previous literature finding (19). These findings may be associated with the ultramild acidity of the adhesive system (pH=2,7), which can affect its interaction with the thick smear layer and does not result in an authentic hybrid layer (20). Furthermore, the high viscosity of the self-etching cement can also affect the flow and wettability of the root dentin substrate (18,21). However, RelyX Ultimate (G4) showed BS values similar to ARC (G1) in all root thirds.

The solvents found in adhesives also play an important role in monomer impregnation, as they can reduce the viscosity of the material and increase the rate of replacement of water, thus facilitating the displacement of water within the demineralized collagen fibrils (22-24). Although the adhesives tested have ethanol as solvent (Adper Sinlge Bond 2/Relyx ARC; Single Bond Universal/ RelyX Ultimate Conventional and Ambar/All Cem) different behaviours were observed. The presence of esters derived from methacrylic acid in the composition of the Single Bond 2 and Single Bond Universal adhesives could explain the better performance of these materials. These esters, due to their acidity, would act as substrate surface reconditioners, improving the performance of adhesives (22). Each system contains a specific functional monomer that determines, among other properties, its retention power. The presence of the 10-MDP functional monomer (10-methacryloyloxidecyl hydrogen phosphate) in the adhesives Single Bond Universal and Ambar, may explain its good performance, because, due to its mild conditioning potential, it would also preserve the hydroxyapatite, serving as a receptor for additional chemical adhesion thus making the bond more stable even in aqueous medium (20).

The results obtained for G2 and G3 may have been influenced by the composition of the respective adhesive systems. In addition to the incorporation of 10-MDP, as already described, G2 presents a silane agent that also contributes to the high values of BS. Silane is a chemical bonding agent, its characteristics being the ability to copolymerize with monomers present in resin cement (25). Furthermore, the cement contains in its formulation a late curing activator for the single bond universal adhesive, which shows better performance when used together, as recommended by the manufacturer.

The similarity between the BS means between the self-adhesive cements and the others reveals that the acidic characteristics of this cement after mixing were sufficient to satisfactorily demineralize the dentin. Although studies report that self-adhesive cement interacts only superficially with dental structures, (19,26), as found in the SEM images of the present study, this interaction was shown to be adequate for satisfactory adherence, highlighting the clinical significance of the technical simplification represented by this material (3,11,24).

Regarding the type of failure, there was no statistically significant differences between cement groups and thirds. The most frequent failures after extrusion tests were mixed failures represented by the association of adhesive and cohesive failure, according to a previous finding in the literature. (27) The failure pattern observed in this study is consistent with the results of the mechanical test, the greater the resistance of the specimen to post-dislocation, the greater the possibility of crack generation, mainly in dentin and cement. In disagreement with previous reports that identified the adhesive failure in the cement-dentin interface as the main failure type (28).

The use of low-resolution SEM was quite adequate for the study of failures, as the resolution (300nm) is sufficient, and sample preparation in this case is not necessary, that is, there is no need for metallization. In addition, due to the low vacuum used in the equipment (compared to the high-resolution SEM), whose system can be opened and closed in 5 minutes, the image is obtained much faster, usually 5-10 min for every 3 samples. Therefore, the images are selected and saved immediately.

To obtain a proper adhesion between dentin and resinous material, a thorough execution of the GFP cementation is necessary. Not only is the luting agent important, but all therapeutic care, since planning, can interfere with the success of rehabilitation with intraradicular retention.

## Conclusions

The self-etching (G4) and self-adhesive (G5) resin cements compared to conventional resin cements (G1, G2 and G3) showed similar results of immediate BS for cementing GFP. Conventional resin cements presented a more uniform hybrid layer with a greater number of resin tags compared to self-etching and self-adhesive resin cements. There was predominance of mixed type failure after push-out tests for all tested materials. Achieving the highest BS values between cement and GFP depends on the selection of materials and their interaction.
